# Comparing Learning Outcomes of Machine-Guided Virtual Reality–Based Training With Educator-Guided Training in a Metaverse Environment: Randomized Controlled Trial

**DOI:** 10.2196/58654

**Published:** 2024-08-07

**Authors:** Dilek Kitapcioglu, Mehmet Emin Aksoy, Arun Ekin Ozkan, Tuba Usseli

**Affiliations:** 1 Center of Advanced Simulation and Education (CASE) Acibadem Mehmet Ali Aydinlar University Istanbul Turkey; 2 Department of Biomedical Device Technology Acibadem Mehmet Ali Aydinlar University Istanbul Turkey; 3 Vocational School for Anaesthesiology Technicians Acibadem Mehmet Ali Aydinlar University Istanbul Turkey

**Keywords:** metaverse, serious gaming, virtual reality, educator guidance, educator, learning, machine guided, VR, guided training, randomized controlled trial, mixed reality, training, training module, module, correlation, gaming, gaming module, serious game, game, games

## Abstract

**Background:**

Virtual reality (VR) modules are commonly used for health care training, such as adult advanced cardiac life support (ACLS), due to immersion and engagement. The metaverse differs from current VR serious gaming by enabling shared social connections, while current VR modules focus on computer-based content without social interaction. Educators in the metaverse can foster communication and collaboration during training sessions.

**Objective:**

This study aimed to compare learning outcomes of VR-based, machine-guided training with educator-guided, VR-based training in the metaverse environment.

**Methods:**

A total of 62 volunteered students from Acibadem Mehmet Ali Aydinlar University Vocational School for Anesthesiology were randomly divided into 2 groups of 31 participants each: one group received VR-based training with machine guidance (MG), and the other received VR-based training with educator guidance (EG) in the metaverse. The members of both groups undertook VR-based basic training for ACLS. Afterward, the MG group was trained with a VR-based advanced training module, which provides training with full MG, whereas the EG group attended the VR-based, educator-guided training in the metaverse. The primary outcome of the study was determined by the exam score of the VR-based training module. Descriptive statistics defined continuous variables such as VR exam scores and time spent on machine- or educator-guided training. The correlation between training time and VR exam scores was assessed with the Spearman rank correlation, and nonnormally distributed variables were compared using the Mann-Whitney *U* test. Statistical significance was set at *P*<.05, with analyses executed by MedCalc Statistical Software (version 12.7.7).

**Results:**

Comparing the VR test scores between the MG and EG groups revealed no statistically significant difference. The VR test scores for the EG group had a median of 86 (range 11-100). In contrast, the MG group scores had a median of 66 (range 13-100; *P*=.08). Regarding the correlation between the duration of machine-guided or educator-guided training and VR-based exam scores, for the MG group, =0.569 and *P*=.005 were obtained. For the EG group, this correlation was found to be =0.298 and *P*=.10. While this correlation is statistically significant for the MG group, it is not significant for the EG group. The post hoc power analysis (80%), considering the correlation between the time spent on training and exam scores, supported this finding.

**Conclusions:**

The results of this study suggest that a well-designed, VR-based serious gaming module with MG could provide comparable learning outcomes to VR training in the metaverse with EG for adult ACLS training. Future research with a larger sample size could explore whether social interaction with educators in a metaverse environment offers added benefits for learners.

**Trial Registration:**

ClinicalTrials.gov NCT06288087; https://clinicaltrials.gov/study/NCT06288087

## Introduction

Serious games are now widely used for training as an additional modality to simulation-based education by various industries [1-5]. Training using serious gaming modules can be conducted through various platforms, such as personal computers; tablet personal computers; virtual reality (VR); or mixed reality systems, such as augmented reality or augmented virtuality. Due to the immersive effect and enhanced student engagement, VR and mixed reality modules are currently favored for health care training, such as advanced cardiac life support (ACLS) training [6-8]. The COVID-19 pandemic has also accelerated the process of digital learning [9]. The shift from conventional in-person classrooms to online education has been expedited by the global pandemic, underscoring the importance of maintaining physical distancing during these training sessions [10-12].

The portability and increased affordability of VR and mixed reality systems in recent years have contributed to a significant growth in VR-based learning. A notable advantage of a VR-based serious gaming module is its compatibility with wireless VR headsets, eliminating the need for a personal computer and an expensive cable-based VR headset [13,14].

Due to advancements in the internet connection speed, the organization of VR-based multiplayer training sessions has become feasible. This facilitates learners’ collaborative interaction within a group setting to solve medical cases [10,15,16].

Online education stands as one of the potential application areas of the metaverse. The metaverse, which is an immersive, 3D, computer-based, and multiuser online environment, serves as an optimal platform for orchestrating multiplayer training sessions [17,18]. The metaverse merges the physical and computer-based environments by integrating the internet, the web, mixed reality, augmented reality, and VR [10].

The metaverse offers shared social connections, thus differentiating it from existing VR-based serious gaming modules, which primarily focus on presenting computer-based content and environments without fostering communal interaction [19]. This facilitates interaction between learners and educators during metaverse-based training sessions, allowing educators to guide the learners and provide feedback [19,20]. The presence of the educators fosters communication and collaboration during training sessions, thereby enhancing the motivation and engagement of the learners [16,21]. While the metaverse has the potential to provide collaborative and individualized learning experiences, the presence of an educator is crucial in guiding and enhancing the learning process [20,22]. Educator-guided learning in the metaverse has the potential to be superior compared with machine-guided learning, as educators can provide personalized feedback, facilitate discussions, and adapt the learning experience to the unique needs and preferences of each student, whereas machine-guided learning can be particularly beneficial for self-learning, where a fixed learning content is available [10,22].

In our university, a VR-based multiplayer training module within the metaverse environment specifically designed for advanced cardiac life support (ACLS) training has been developed [23]. This module serves as an additional training tool to simulation-based training, enabling learners to receive guidance either from machine algorithms or educators within the metaverse. In light of the limitations of machine-guided learning in VR for collaborative learning and the time constraints associated with educator-guided training in a metaverse environment, a comparative analysis of these methods has been deemed necessary [10,22,24]. The hypothesis of this research is to evaluate and contrast the learning outcomes achieved through VR-based, machine-guided training against educator-guided, VR-based training within the metaverse setting.

## Methods

### Recruitment

The study was advertised to fourth-semester (2023-2024 Spring Semester) students at the Vocational School for Anesthesiology at Acibadem Mehmet Ali Aydinlar University, and volunteers were accepted for participation. In total, 62 participants volunteered for the study, including 36 female participants and 26 male participants between 20 and 22 years of age. Participants were randomized into 2 groups based on their university ID numbers, with students being assigned to one group if their ID number was odd and to the other group if their ID number was even. By using this randomization process, the participants were divided into 2 groups, each consisting of 31 individuals: one group received VR-based training with machine guidance (MG), and the other group received VR-based training with educator guidance (EG) in the metaverse. All participants read and signed an informed consent document outlining the study flow. The study took place at the Center of Advanced Simulation and Education of Acibadem Mehmet Ali Aydinlar University. Exclusion criteria included previously experienced VR-induced motion sickness; previous ACLS training; and other medical conditions, such as episodes of vertigo or being on medication that causes vertigo-like symptoms.

### Study Flow

From this point forward, the group trained with VR-based training with MG will be referred to as the MG group and the group trained with VR-based training with EG in metaverse will be referred to as the EG group. A total of 2 educators with more than 10-year experience in medical simulation guided the participants in metaverse during the study.

The members of both groups undertook VR-based basic training for ACLS. Afterward, participants of the MG group were trained with a VR-based advanced training module, which provides training with full MG, whereas the EG group attended VR-based, educator-guided training in the metaverse.

### Serious Gaming Module

The VR-based serious game used in this study, named “3DMedsim ACLS VR,” was developed in line with the ACLS standards of the European Resuscitation Council (ERC) and American Heart Association (AHA) [25,26]. The development process was reviewed by clinicians to ensure adherence to crisis resource management and the criteria of the latest AHA and ERC guidelines [27-29].

The serious game is combined with a learning management system along with a learning record store (LRS), allowing the storage of credentials of the users within a shared database [30-32]. In addition, it incorporates a 3D visualization engine integrated with the LRS, enabling the tracking of user interactions and the generation of experience application programming interface (xAPI) calls for each action taken [31]. When users engage in predefined interactions with significant objects within the computer-based environment, corresponding xAPI events are automatically generated [30,33]. This functionality is made possible through the development of a Unity extension software library making necessary xAPI web service calls to the LRS servers through the HTTP protocol [34]. User actions are defined in terms of actor, verb, and object parameters, with the library automatically creating xAPI calls for each action, subsequently recorded by the LRS. The library is also capable of automatically creating xAPI calls for actions not performed within specified time limits or in the correct sequence. Security measures and user authentication features essential for record-keeping are also included in this library, using the basic access authentication method inherent in the http protocol standard.

**Figure 1:**
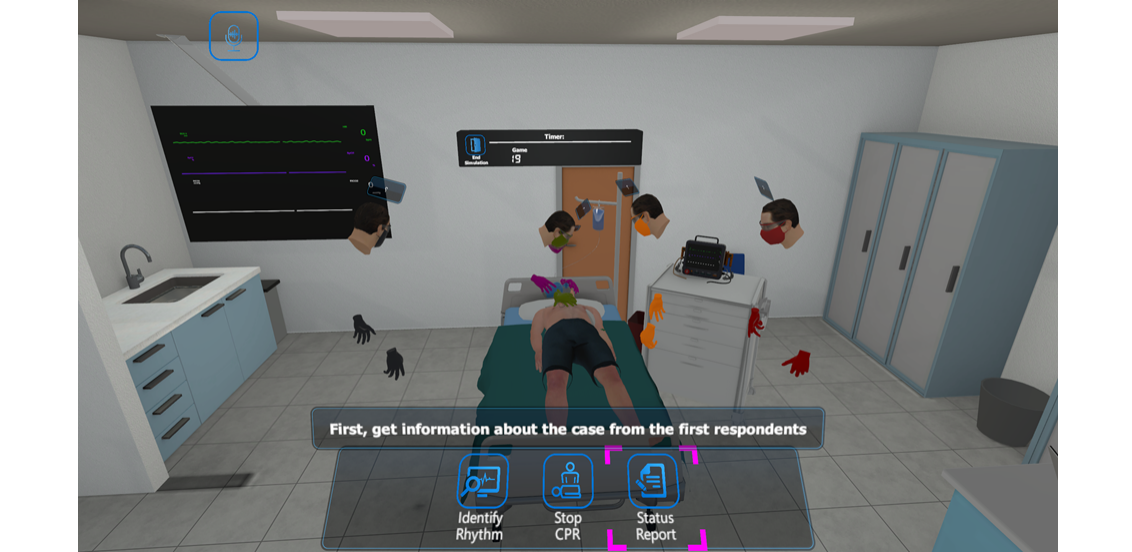
A computer-based hospital room setting in the serious gaming module, including selection options for the next step in the advanced cardiac algorithm. CPR: cardiopulmonary resuscitation.

The ACLS serious game includes 4 different stages: basic training, advanced training with machine support, advanced training with educator support in the metaverse environment, and test mode. All of these stages take place in a computer-based hospital room setting to increase immersion, as shown in Figure 1. This serious game also allows users to create a multiplayer lobby and invite others as observers to join the training. These multiplayer lobbies include a voice chat for communication between the observers and the player [13].

In the first stage, basic training is developed with complete beginners in mind. This stage aims to walk the player through the ACLS algorithm step by step with visual and audio cues for each interaction. The user is only able to interact with correct objects in this stage, with no time limits, until they felt ready for advanced-level training mode. The removal of timings and wrong choices removes the stress element and allows the user to familiarize themselves with the ACLS algorithm [13].

**Figure 2:**
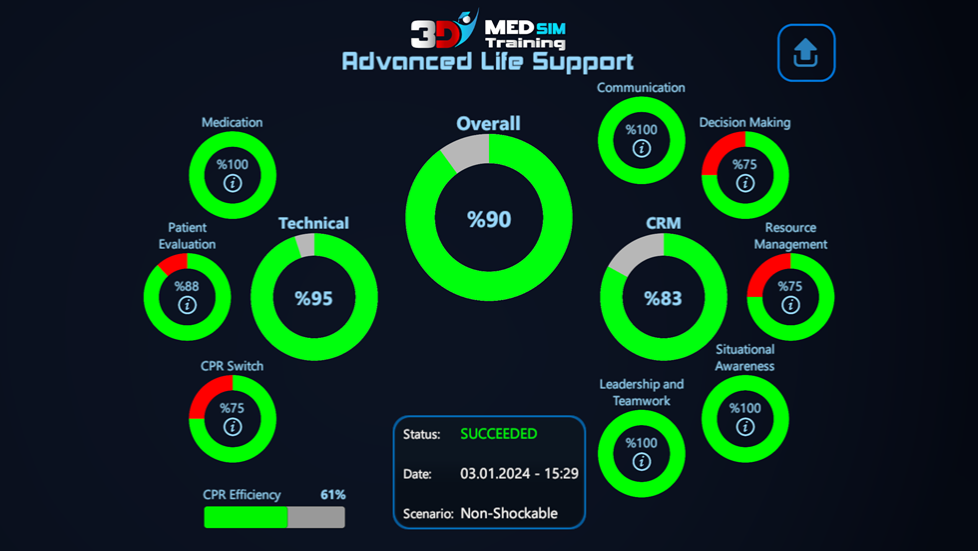
The score screen of the advanced cardiac life support serious game exam mode, including separate scores for technical skills, nontechnical skills, and the total score. CPR: cardiopulmonary resuscitation.

In the second stage, advanced training builds on top of basic training. In this stage, visual and audio help for each step is removed. The user is also able to interact with any object or button, which means making mistakes is a possibility at this stage. However, if the user makes a mistake, they are warned by MG. The system monitors each action performed by the player and generates verbal warnings when errors are detected or if time limits are exceeded. In the study, the MG group played this mode independently with MG, while the EG group played the mode in the metaverse environment, accompanied by an educator, as depicted in Figure 2. In educator-guided mode, the system disables machine-guided warnings, enabling educators to provide real-time guidance during training in the metaverse [13].

**Figure 3:**
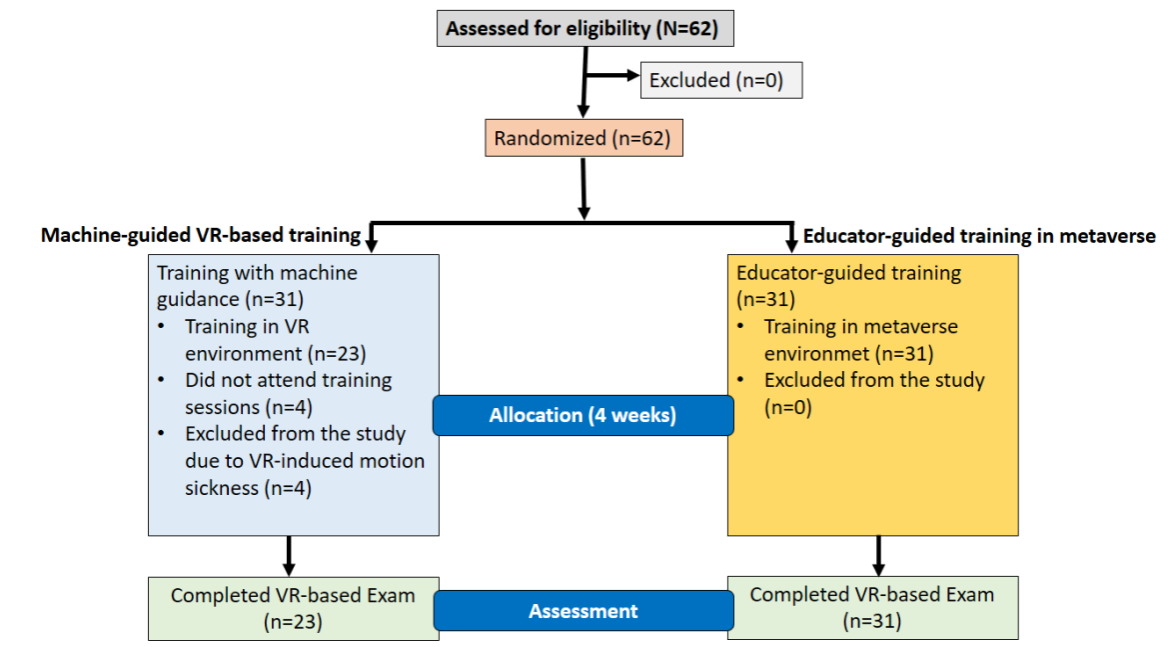
Randomized controlled trial flow diagram of the study. VR: virtual reality.

The final stage is the VR-based exam mode, which was completed by participants immediately following the MG and EG training modes on the same day. In this mode, each action taken by the user is graded according to its timing, order, and accuracy. Contrary to the advanced training mode, there was no guidance for the users. The final score consisted of 2 categories. In total, 70% (70/100) of the total performance score is derived from ACLS assessments, with the remaining percentage (30/100, 30%) calculated from crisis resource management performances of the participants. The primary outcome of the study was determined by the total score obtained through the exam mode of the VR-based training module [13]. A sample test score report can be seen in Figure 3.

### Data Collection

The mean test scores obtained by participants in the VR-based training module exam, along with the duration of machine-guided or educator-guided training (in minutes), were chosen as key indicators for the outcomes if the study. The correlation between exam scores and time spent on training was investigated.

### Statistical Analysis

Descriptive statistics were used to define continuous variables, that is, VR exam scores and time spent for machine- or educator-guided training (minutes; mean, SD, median, and range). The correlation between time spent for machine- or educator-guided training and VR exam scores that did not show normal distribution was evaluated using the Spearman rank correlation. The comparison of MG and EG groups that did not show normal distribution in terms of VR exam score and time spent for machine- or educator-guided training was performed using the Mann-Whitney *U* test. The statistical significance level was set at *P*=.05. Analyses were conducted using MedCalc Statistical Software (version 12.7.7) [35].

### Ethical Considerations

This study was approved by the Scientific Ethical Committee of Acibadem Mehmet Ali Aydinlar University (approval 2023-20/672). All participants read and signed an informed consent document outlining the study flow. No secondary analysis was performed using these data (Multimedia Appendices 1 and 2).

The data used in the research do not contain any information that can identify individuals. Each participant was assigned a unique code that replaced personal identifiers. The key linking codes to personal information were securely stored separately from the research data. Only essential research team members have access to the deidentified data. Participants did not receive any financial compensation. The participants were informed with the consent form that the results of this research may be used in scientific and professional publications or for educational purposes, but the identities of the participants will remain confidential. The relevant consent form (Turkish or English) can be seen in Multimedia Appendices 1 and 2.

## Results

### Overview

There were 4 dropouts from the MG group because they did not meet the inclusion criteria. In addition to this, 4 participants from the MG group did not attend the training sessions. Therefore, the EG consisted of 31 participants, whereas the MG group consisted of 23 participants (Figure 3 and Multimedia Appendix 3).

After undertaking a VR-based basic training for ACLS, the participants were divided into 2 groups, MG (n=23) and EG (n=31). The participants of the MG group were trained with a VR-based advanced training module with full MG. The participants of the EG group attended a VR-based, educator-guided training in the metaverse environment. In order to evaluate the knowledge level of the participants, the exam mode of the VR-based training module was used.

As shown in Table 1, the average score was 53 (SD 33) out of 100 for the MG group participants and 68 (SD 35) out 100 for the EG group participants. When comparing the VR test scores, no significant difference was found between the MG and EG groups. The average VR test scores for the EG group were 68 (SD 35), with a median of 86 (range 11-100), whereas MG group scores were 53 (SD 33), with a median of 66 (range 13-100; *P*=.08), as seen in Table 1.

**Table 1:** Mean and median scores of educator guidance and machine guidance group participants.

Virtual reality exam score (total 100 points)	Machine guidance group	Educator guidance group	*P* value^a^
Mean (SD)	53 (33)	68 (35)	.08
Median (range)	66 (13-100)	86 (11-100)	–^b^

^a^*P*<.005 for all exam scores.

^b^Not applicable.

### Mann-Whitney U Test

After comparing the time spent (in minutes) for machine-guided training and educator-guided training for each participant, a significant difference was found between the MG and EG groups. The time spent in the EG group had a median of 21 (range 14-62) minutes. In contrast, the MG group had a median of 15 (range 5-30) minutes (*P*=.002).

### Correlation Between VR Test Scores and Duration of the Training

Regarding the correlation between the duration of machine- or educator-guided training and VR test scores, for the MG group, =0.569 and *P*=.005 were obtained. For the EG group, this correlation was found to be =0.298 and *P*=.10. While this correlation is statistically significant for the MG group, it is not significant for the EG group.

### Spearman Rank Correlation Test

If the power analysis is based on the exam scores of the 2 groups, the type I error rate was found to be 40% at a 5% significance level. However, when a post hoc power analysis was conducted, considering the correlation between the time spent on training and exam scores, the type I error rate increased to 80% at the same significance level [36,37].

## Discussion

### Principal Findings

This study focuses on comparing the learning outcomes of machine- versus educator-guided, VR-based training in the metaverse environment. Participants of the EG and MG groups were evaluated using the exam mode of the VR-based training module, which was defined as the main outcome indicator of this study. The findings of the study revealed that there was no statistical difference between the VR-based exam scores of the MG and EG groups. Although these values are statistically significant, the post hoc power analysis based on the exam scores of the 2 groups indicated a power of only 40%. Since this is a preliminary study, a power analysis was not conducted beforehand. While the results are statistically valid, they might be influenced by insufficient power. From an educational perspective, however, the observed difference appears relevant. In addition to this finding, the participants of the EG group spent more time during the advanced training compared with the participants of the MG group (*P*=.002). The correlation between the duration of machine- or educator-guided training and VR-based exam scores is statistically significant for the MG group (=0.569, *P*=.005), but not significant for the EG group (=0.298, *P*=.10). These findings indicate that dedicating additional time to VR-based, machine-guided training holds promise for boosting exam scores of the participants. The post hoc power analysis (80%), considering the correlation between the time spent on training and exam scores, supported this finding.

Using well-designed, VR-based serious gaming modules for specific tasks can enhance learners’ motivation, resulting in improved learning outcomes [9,10,16,19,22,38]. The serious gaming module used in this study, specifically designed for adult ACLS training, has proven its capability of improving the knowledge level during our previous study [13].

Numerous studies have demonstrated that VR-based training within the metaverse environment enhances learning outcomes, attributed to its immersive effects and interactive features [15,20,38-41]. Interactivity enables users to engage and communicate through avatars, enhancing embodiment, immersion, and user engagement within the metaverse [9,10,20,39]. The training module used in this study provides different real-life hospital environments to enhance the immersive effect and multiplayer mode to enable users to interact with each other.

A gamified interaction may experience insufficient supervision. The presence of an educator in the metaverse has the potential to enhance learning outcomes, particularly for learners who struggle with self-regulation [9,21]. Introducing multiplayer features within the metaverse can address this limitation, fostering social interaction and potentially enhancing learning outcomes further [19,20]. However, performance data gathered during this study indicated that there was no statistical difference in the exam scores between the MG and EG groups, suggesting that EG did not provide any additional advantage in terms of achieving better learning outcomes.

According to previous studies, an important limitation of metaverse-based learning is the lack of sufficient metaverse literacy among both learners and educators, revealing the importance of familiarization with effective metaverse usage [19,24]. As VR-based serious gaming modules have been embedded into the curricula of training programs of our university for the last 6 years, the students and educators, who have participated in this study, did not encounter any issues with the usage of VR hardware.

Widespread adoption of VR hardware is still restricted among a notable portion of the population. This problem is exacerbated by the lack of high-speed internet connectivity in certain regions of the world. [11,42]. As the same problem also exists in our country, this study was performed on the university campus with high-speed internet access and the VR headsets were provided by the simulation center of our university.

### Limitations

The first limitation of the study was the small sample size, which was attributed to the challenges faced in recruiting volunteers. The second limitation of the study was the limited processing power and graphics capability of the headsets used in this study. Therefore, only the heads of the avatars were displayed during the game, leading to a decreased level of immersion. The third limitation of this study was the potential for selection bias. Participants were recruited from a specific population, which may not represent the broader demographic. This can limit the generalizability of our findings. In addition, the voluntary nature of participation may attract individuals with particular characteristics or interests, further skewing the results. Future studies should aim for a more diverse and random sample to mitigate this bias.

### Conclusions

Based on the findings of this study, a properly designed VR-based serious gaming module with MG could potentially offer similar learning outcomes to VR-based training in the metaverse with EG. Future research with a larger sample size could explore whether the effect of social interaction with educators in the metaverse environment may provide additional benefits for learners.

## Appendix

Multimedia Appendix 1 Consent form for the study (Turkish Version).

Multimedia Appendix 2 Consent form for the study (English Version).

Multimedia Appendix 3 CONSORT-eHEALTH checklist (V 1.6.1).

Multimedia Appendix 4 Raw data of the study.

## Abbreviations

ACLS: advanced cardiac life support
AHA: American Heart Association
EG: educator guidance
ERC: European Resuscitation Council
LRS: learning record store
MG: machine guidance
VR: virtual reality
xAPI: experience application programming interface

## Data Availability

All data generated or analyzed during this study are included in this published paper and Multimedia Appendix 4.
