# Supplementary material for: Comparing Learning Outcomes of Machine-Guided Virtual Reality–Based Training With Educator-Guided Training in a Metaverse Environment: Randomized Controlled Trial
Source: JMIR Serious Games. 2024 Aug 7;12:e58654. doi: 10.2196/58654 (PMC11339586; doi:10.2196/58654)
Supplement: Multimedia Appendix 1 [file games_v12i1e58654_app1.pdf]

Aralık 2023

ARAŞTIRMAYA GÖNÜLLÜ KATILIM FORMU

Bu çalışma Acıbadem Üniversitesi Tıp Eğitimi Bölümü öğretim üyelerinden Dr.Öğr.Üyesi Dilek Kitapçıoğlu tarafından yürütülmektedir. Bu form sizi araştırma koşulları hakkında bilgilendirmek için hazırlanmıştır.

Çalışmanın Amacı Nedir?

Metaverse ortamında ileri yaşam desteği eğitiminde bilgisayar yönlendirmeli modül ile eğitici yönlendirmeli modülün etkinliğinin karşılaştırılması amaçlanmıştır.

Bize Nasıl Yardımcı Olmanızı İsteyeceğiz?

Araştırma CASE Simülasyon Merkezi'nde yapılacaktır. Üniversite öğrencileri katılımcı olarak davet edilecek, katılmak isteyenler yaklaşık 30 dak eğitim sonrasında 10 dak sınava hazırlık eğitimi (bir grup bilgisayar yönlendirmeli diğer grup eğitici yönlendirmeli olmak üzere ve 10 dak sınav olmak üzere sanal gerçeklik ortamında ileri yaşam desteği eğitim modülünü tamamlayacaktır.

Katılımınızla ilgili bilmeniz gerekenler:

Bu çalışmaya katılmak tamamen gönüllülük esasına dayalıdır. Herhangi bir yaptırıma veya cezaya maruz kalmadan çalışmaya katılmayı reddedebilir veya çalışmayı bırakabilirsiniz. Araştırma esnasında cevap vermek istemediğiniz sorular olursa boş bırakabilirsiniz.

Araştırmaya katılanlardan toplanan veriler tamamen gizli tutulacak, veriler ve kimlik bilgileri herhangi bir şekilde eşleştirilmeyecektir. Katılımcıların isimleri bağımsız bir listede toplanacaktır. Ayrıca toplanan verilere sadece araştırmacılar ulaşabilecektir. Bu araştırmanın sonuçları bilimsel ve profesyonel yayınlarda veya eğitim amaçlı kullanılabilir, fakat katılımcıların kimliği gizli tutulacaktır.

Riskler:

Bu çalışmada sanal gerçeklik gözlüğü kullanılacaktır.

Bu yüzden aşağıdaki semptomlardan herhangi birini daha önce geçirdiyseniz araştırmaya katılmanız uygun değildir.

- **Vertigo**
- **Otitis media**
- **Vertigo semptomlarına benzer yan etkisi olan ilaç kullanımı**

Araştırmayla ilgili daha fazla bilgi almak isterseniz:

Çalışmayla ilgili soru ve yorumlarınızı araştırmacıya ***dilek.kitapcioglu@acibadem.edu.tr*** adresinden iletebilirsiniz.

Center of Advanced Simulation and Education

Yukarıdaki bilgileri okudum ve bu çalışmaya tamamen gönüllü olarak katılıyorum.
(Formu doldurup imzaladıktan sonra uygulayıcıya geri veriniz).

İsim Soyad Tarih İmza

---/----/-----
