# Supplementary material for: Comparing Learning Outcomes of Machine-Guided Virtual Reality–Based Training With Educator-Guided Training in a Metaverse Environment: Randomized Controlled Trial
Source: JMIR Serious Games. 2024 Aug 7;12:e58654. doi: 10.2196/58654 (PMC11339586; doi:10.2196/58654)
Supplement: Multimedia Appendix 2 [file games_v12i1e58654_app2.pdf]

VOLUNTARY PARTICIPATION FORM FOR RESEARCH

This study is conducted by Assist.Prof.Dr.Dilek Kitapçıoğlu from the Department of Medical Education at Acıbadem University. This form is prepared to inform you about the research conditions.

What is the Aim of the Study?

The study aims to compare the effectiveness of a computer-directed module and an instructor-directed module in advanced life support education in the Metaverse environment.

How Can You Help Us?

The research will be conducted at CASE Simulation Center. University students will be invited to participate. Those willing to participate will complete approximately 30 minutes of training followed by 10 minutes of exam preparation (one group with computer-directed guidance and the other with instructor-directed guidance), and then a 10-minute exam, all within a virtual reality environment for advanced life support education.

What You Should Know About Your Participation?

Participation in this study is entirely voluntary. You may refuse to participate or withdraw from the study at any time without any penalties or consequences. If there are questions during the research that you do not wish to answer, you may leave them blank.

Data collected from participants will be kept strictly confidential, and data and identity information will not be linked in any way. Participants' names will be kept in an independent list. Additionally, only researchers will have access to the collected data. The results of this research may be used for scientific and professional publications or educational purposes, while maintaining participant anonymity.

Risks

This study involves the use of virtual reality goggles. Therefore, if you have experienced any of the following symptoms before, participating in the research is not advisable: • Vertigo • Otitis media • Use of medications with side effects similar to vertigo symptoms

For more information about the study: You can send your questions and comments about the study to the researcher at dilek.kitapcioglu@acibadem.edu.tr.

I have read the above information and I am voluntarily participating in this study. (After filling out and signing the form, please return it to the practitioner.)

Name **Surname** **Signature**

Date
