# Supplementary material for: Comparing Learning Outcomes of Machine-Guided Virtual Reality–Based Training With Educator-Guided Training in a Metaverse Environment: Randomized Controlled Trial
Source: JMIR Serious Games. 2024 Aug 7;12:e58654. doi: 10.2196/58654 (PMC11339586; doi:10.2196/58654)
Supplement: Multimedia Appendix 3 [file games_v12i1e58654_app3.pdf]

CONSORT-EHEALTH Checklist V1.6.2 Report	Manuscript Number	58654
**(based on CONSORT-EHEALTH V1.6), available at [http://tinyurl.com/consort-ehealth-v1-6].**		
**Date completed**		
4/16/2019 17:36:45		
**by**		
Emin Aksoy		
Comparing the Effects on Learning Outcomes of Tablet-Based and Virtual Reality–Based Serious Gaming Modules for Basic Life Support Training: Randomized Trial		
TITLE		
**1a-i) Identify the mode of delivery in the title**		
Tablet-Based and Virtual Reality–Based Serious Gaming Modules		
**1a-ii) Non-web-based components or important co-interventions in title**		
"Tablet-Based and Virtual Reality–Based Serious Gaming Modules". The gaming modules are using web based apps.		
**1a-iii) Primary condition or target group in the title**		
There is no information mentioning information about the target group.		
ABSTRACT		
**1b-i) Key features/functionalities/components of the intervention and comparator in the METHODS section of the ABSTRACT**		
key features/functionalities/components of the intervention are mentioned in the abstract.		
**1b-ii) Level of human involvement in the METHODS section of the ABSTRACT**		
50<br>first-semester students of Acibadem Mehmet Ali Aydinlar<br>University Vocational School for Paramedics		
**1b-iii) Open vs. closed, web-based (self-assessment) vs. face-to-face assessments in the METHODS section of the ABSTRACT**		
First-semester students of Acibadem Mehmet Ali Aydinlar<br>University Vocational School for Paramedics volunteered to<br>participate in this study. "<br><br><br>"After the BLS training, students in both groups then had to take a posttest to<br>assess their acquired knowledge. The content of the pre- and<br>posttest is shown in Table 1."		
**1b-iv) RESULTS section in abstract must contain use data**		
Yes. study was designed as a randomized trial comparing pretest and posttest results. A tablet-based and VR-based<br>serious game with identical content was used for 40 participants. Over half of them (22/40, 55%) were included in the VR group<br>and just under half (18/40, 45%) were in the tablet group.		
**1b-v) CONCLUSIONS/DISCUSSION in abstract for negative trials**		
No		
INTRODUCTION		
**2a-i) Problem and the type of system/solution**		
As the paper focuses on teaching "Basic Life Support", the target group for teaching basic life support is very big.		
**2a-ii) Scientific background, rationale: What is known about the (type of) system**		
In recent decades, new educational techniques have been adapted<br>to address the constantly changing needs and expectations of<br>educators and learners due to advances in technology. A reason<br>for this shift was that students traditionally must learn the same<br>thing at the same time, with the result that classes and training<br>sessions are frustratingly hard for some and too easy for others		
**Does your paper address CONSORT subitem 2b?**		
The aim of this study is to compare the effect of the knowledge<br>level of participants after using the tablet-based serious game<br>for BLS and the VR-based serious game for BLS with the help<br>of a pretest and a posttest.		
METHODS		
**3a) CONSORT: Description of trial design (such as parallel, factorial) including allocation ratio**		
The aim of this study is to compare the effects on the knowledge level of participants after using a tablet-based<br>serious game and a virtual reality (VR)–based serious game for Basic Life Support using a pretest/posttest method.		
**3b) CONSORT: Important changes to methods after trial commencement (such as eligibility criteria), with reasons**		
No		
**3b-i) Bug fixes, Downtimes, Content Changes**		
**4a) CONSORT: Eligibility criteria for participants**		
No		
**4a-i) Computer / Internet literacy**		
A very important risk factor of the VR version was the potential<br>problem of dizziness. Similar to motion sickness, VR sickness<br>is caused due to mismatch between the visual and vestibular<br>systems. VR-based serious game scenarios must take this risk<br>into account. Participants were warned about this risk on their<br>written consent forms before using the VR-based version. The<br>VR-based version of the serious game was used in a special<br>room with soft flooring material and special walls covered with<br>soft material in order to minimize the risk of trauma in case of<br>dizziness problems.		
**4a-ii) Open vs. closed, web-based vs. face-to-face assessments:**		
The participants were recruited on voluntary basis among the first semester paramedic students taking part at medical simulation session at our university.		
**4a-iii) Information giving during recruitment**		
"The participants were informed about the study and filled out<br>consent forms. "		
**4b) CONSORT: Settings and locations where the data were collected**		
Yes, Acibadem Mehmet Ali Aydinlar University, Center of Advanced Simulation and Education, Istanbul, Turkey		
**4b-i) Report if outcomes were (self-)assessed through online questionnaires**		
The outcomes were assessed by using online questionnaires.		
**4b-ii) Report how institutional affiliations are displayed**		
The participants were first semester paramedic students of our university volunteering to take part in the study.		
**5) CONSORT: Describe the interventions for each group with sufficient details to allow replication, including how and when they were actually administered**		

**5-i) Mention names, credential, affiliations of the developers, sponsors, and owners**		
Yes.		
**5-ii) Describe the history/development process**		
No		
**5-iii) Revisions and updating**		
No		
**5-iv) Quality assurance methods**		
No		
**5-v) Ensure replicability by publishing the source code, and/or providing screenshots/screen-capture video, and/or providing flowcharts of the algorithms used**		
Screen captures of the VR base and Tablet PC based of the gaming modules are included.		
**5-vi) Digital preservation**		
NO		
**5-vii) Access**		
No		
**5-viii) Mode of delivery, features/functionalities/components of the intervention and comparator, and the theoretical framework**		
No		
**5-ix) Describe use parameters**		
The participants were informed about the study and filled out consent forms. A very important risk factor of the VR version was the potential problem of dizziness. Similar to motion sickness, VR sickness is caused due to mismatch between the visual and vestibular systems. VR-based serious game scenarios must take this risk into account. Participants were warned about this risk on their written consent forms before using the VR-based version.		
**5-x) Clarify the level of human involvement**		
50 first-semester students of Acibadem Mehmet Ali Aydinlar University Vocational School for Paramedics volunteered to participate in this study. The participants (N=50) were randomly divided into two groups with 25 participants each. Since we had some dropouts due to personal reasons, we ended up with 22 of 25 (88%) participants in the VR group and 18 of 25 (72%) in the tablet group using the tablet version of our serious game.		
**5-xi) Report any prompts/reminders used**		
No		
**5-xii) Describe any co-interventions (incl. training/support)**		
None of the participants received any prior education about the ERC 2015 BLS algorithm and had no prior VR experience.		
**6a) CONSORT: Completely defined pre-specified primary and secondary outcome measures, including how and when they were assessed**		
Students from both groups had to complete a pretest to assess their prior knowledge on BLS procedures. After the BLS training, students in both groups then had to take a posttest to assess their acquired knowledge. The content of the pre- and posttest is shown in Table 1. The difference between the preand posttest was the scrambled order of questions and answers		
**6a-i) Online questionnaires: describe if they were validated for online use and apply CHERRIES items to describe how the questionnaires were designed/deployed**		
Students from both groups had to complete a pretest to assess their prior knowledge on BLS procedures. After the BLS training, students in both groups then had to take a posttest to assess their acquired knowledge. The content of the pre- and posttest is shown in Table 1. The difference between the preand posttest was the scrambled order of questions and answers		
**6a-ii) Describe whether and how “use” (including intensity of use/dosage) was defined/measured/monitored**		
No		
**6a-iii) Describe whether, how, and when qualitative feedback from participants was obtained**		
No		
**6b) CONSORT: Any changes to trial outcomes after the trial commenced, with reasons**		
Yes, Acibadem Mehmet Ali Aydinlar University, Center of Advanced Simulation and Education, Istanbul, Turkey		
**7a) CONSORT: How sample size was determined**		
**7a-i) Describe whether and how expected attrition was taken into account when calculating the sample size**		
No		
**7b) CONSORT: When applicable, explanation of any interim analyses and stopping guidelines**		
Students from both groups had to complete a pretest to assess their prior knowledge on BLS procedures. After the BLS training, students in both groups then had to take a posttest to assess their acquired knowledge. The content of the pre- and posttest is shown in Table 1. The difference between the preand posttest was the scrambled order of questions and answers		
**8a) CONSORT: Method used to generate the random allocation sequence**		
No		
**8b) CONSORT: Type of randomisation; details of any restriction (such as blocking and block size)**		
No		
**9) CONSORT: Mechanism used to implement the random allocation sequence (such as sequentially numbered containers), describing any steps taken to conceal the sequence until interventions were assigned**		
No		
**10) CONSORT: Who generated the random allocation sequence, who enrolled participants, and who assigned participants to interventions**		
No		
**11a) CONSORT: Blinding - If done, who was blinded after assignment to interventions (for example, participants, care providers, those assessing outcomes) and how**		
**11a-i) Specify who was blinded, and who wasn’t**		
No		
**11a-ii) Discuss e.g., whether participants knew which intervention was the “intervention of interest” and which one was the “comparator”**		
No		
**11b) CONSORT: If relevant, description of the similarity of interventions**		
he 3DMedSim tablet-based BLS serious gaming app and 3DMedSim VR-based BLS serious gaming module identical content and difficulty level were used for this study.		
**12a) CONSORT: Statistical methods used to compare groups for primary and secondary outcomes**		

Statistical analysis was performed using the MedCalc Statistical Software version 12.7.7 [9]. For comparison of two non-normally distributed dependent groups, Wilcoxon signed rank test was used. As seen in Table 2, there is a significant difference between average pre- and posttest results in both groups (P<.05). In order to calculate the effect size of the results, Cohen d has been used.

12a-i) Imputation techniques to deal with attrition / missing values

No

12b) CONSORT: Methods for additional analyses, such as subgroup analyses and adjusted analyses

No

RESULTS

13a) CONSORT: For each group, the numbers of participants who were randomly assigned, received intended treatment, and were analysed for the primary outcome

Yes.

13b) CONSORT: For each group, losses and exclusions after randomisation, together with reasons

The participants (N=50) were randomly divided into two groups with 25 participants each. Since we had some dropouts due to personal reasons, we ended up with 22 of 25 (88%) participants in the VR group and 18 of 25 (72%) in the tablet group using the tablet version of our serious game.

13b-i) Attrition diagram

No.

14a) CONSORT: Dates defining the periods of recruitment and follow-up

No

14a-i) Indicate if critical “secular events” fell into the study period

No

14b) CONSORT: Why the trial ended or was stopped (early)

The participants (N=50) were randomly divided into two groups with 25 participants each. Since we had some dropouts due to personal reasons, we ended up with 22 of 25 (88%) participants in the VR group and 18 of 25 (72%) in the tablet group using the tablet version of our serious game.

15) CONSORT: A table showing baseline demographic and clinical characteristics for each group

This data was mentioned in the main text " 50 first-semester students of Acibadem Mehmet Ali Aydinlar University Vocational School for Paramedics volunteered to participate in this study. The participants (N=50) were randomly divided into two groups with 25 participants each. Since we had some dropouts due to personal reasons, we ended up with 22 of 25 (88%) participants in the VR group and 18 of 25 (72%) in the tablet group using the tablet version of our serious game. The participants were informed about the study and filled out consent forms. None of the participants received any prior education about the ERC 2015 BLS algorithm and had no prior VR experience. "

15-i) Report demographics associated with digital divide issues

No.

16a) CONSORT: For each group, number of participants (denominator) included in each analysis and whether the analysis was by original assigned groups

16-i) Report multiple “denominators” and provide definitions

No

16-ii) Primary analysis should be intent-to-treat

17a) CONSORT: For each primary and secondary outcome, results for each group, and the estimated effect size and its precision (such as 95% confidence interval)

Yes. "Students from both groups had to complete a pretest to assess their prior knowledge on BLS procedures. After the BLS training, students in both groups then had to take a posttest to assess their acquired knowledge. The content of the pre- and posttest is shown in Table 1. The difference between the preand posttest was the scrambled order of questions and answers"

17a-i) Presentation of process outcomes such as metrics of use and intensity of use

No

17b) CONSORT: For binary outcomes, presentation of both absolute and relative effect sizes is recommended

No

18) CONSORT: Results of any other analyses performed, including subgroup analyses and adjusted analyses, distinguishing pre-specified from exploratory

No.

18-i) Subgroup analysis of comparing only users

Yes. " For comparison of two non-normally distributed dependent groups, Wilcoxon signed rank test was used. As seen in Table 2, there is a significant difference between average pre- and posttest results in both groups (P<.05). In order to calculate the effect size of the results, Cohen d has been used [10]. The effect size of the VR results are significantly higher compared with the tablet version. Mean posttest results were significantly higher in both groups. The difference between the pre- and posttest results were significantly higher in the VR group, and these data were statistically significant (Student t test P<.05) as seen in Table 3 and Figure 3."

19) CONSORT: All important harms or unintended effects in each group

Although there was a risk of feeling dizzy while using the VR-based version, this problem was not encountered during this study. Since we used the highest flickering rate and screen resolution with today’s technology, we did not encounter any kind of clinical problems such as dizziness or headache during VR training.

19-i) Include privacy breaches, technical problems

No

19-ii) Include qualitative feedback from participants or observations from staff/researchers

No

DISCUSSION

20) CONSORT: Trial limitations, addressing sources of potential bias, imprecision, multiplicity of analyses

20-i) Typical limitations in ehealth trials

One the limitations in our study was that the participants were not familiar with using VR-based systems in the beginning of the study compared to the group using tablet PCs for the serious gaming app. Due to widespread use of tablets and mobile phones in the Turkish population, participants encountered no difficulties using the tablet-based app. VR-based systems are rather new technologies with very few people having access to this technology at this time in our country.

**21) CONSORT: Generalisability (external validity, applicability) of the trial findings**		
**21-i) Generalizability to other populations**		
We contend that VR-based serious gaming provides interactivity and a higher level of presence due to higher immersion levels, therefore it is already being widely used for training purposes in many areas. But there are only a few apps being used in medical education. Despite this, we believe that VR-based serious gaming modules will have many areas of application in medical education as well. Our study indicates that serious gaming has a positive effect on the learning outcome of digital learners.		
**21-ii) Discuss if there were elements in the RCT that would be different in a routine application setting**		
No		
**22) CONSORT: Interpretation consistent with results, balancing benefits and harms, and considering other relevant evidence**		
**22-i) Restate study questions and summarize the answers suggested by the data, starting with primary outcomes and process outcomes (use)**		
Despite this, we believe that VR-based serious gaming modules will have many areas of application in medical education as well. Our study indicates that serious gaming has a positive effect on the learning outcome of digital learners.		
**22-ii) Highlight unanswered new questions, suggest future research**		
Further studies need to be performed on the effectiveness of serious gaming in health care training.		
Other information		
**23) CONSORT: Registration number and name of trial registry**		
(JMIR Serious Games 2019;7(2):e13442) doi:10.2196/13442		
**24) CONSORT: Where the full trial protocol can be accessed, if available**		
No		
**25) CONSORT: Sources of funding and other support (such as supply of drugs), role of funders**		
There no funding sources		
**X26-i) Comment on ethics committee approval**		
After approval by the Ethical Committee of Acibadem Mehmet Ali Aydinlar University, 50 first-semester students of Acibadem Mehmet Ali Aydinlar University Vocational School for Paramedics volunteered to participate in this study.		
**x26-ii) Outline informed consent procedures**		
Yes, the participants were informed about the study and filled out consent forms.		
**X26-iii) Safety and security procedures**		
A very important risk factor of the VR version was the potential problem of dizziness. Similar to motion sickness, VR sickness is caused due to mismatch between the visual and vestibular systems. VR-based serious game scenarios must take this risk into account. Participants were warned about this risk on their written consent forms before using the VR-based version. The VR-based version of the serious game was used in a special room with soft flooring material and special walls covered with soft material in order to minimize the risk of trauma in case of dizziness problems.		
**X27-i) State the relation of the study team towards the system being evaluated**		
None declared		
