# Supplementary material for: Comparing Learning Outcomes of Machine-Guided Virtual Reality–Based Training With Educator-Guided Training in a Metaverse Environment: Randomized Controlled Trial
Source: JMIR Serious Games. 2024 Aug 7;12:e58654. doi: 10.2196/58654 (PMC11339586; doi:10.2196/58654)
Supplement: Multimedia Appendix 4 [file games_v12i1e58654_app4.pdf]

Machine Guided Group of Participants

Participant	time spent	VR Exam Score
ALS 26	58	66
ALS 29	46	82
ALS 30	53	92
ALS 31	62	73
ALS 28	45	48
ALS 23	49	69
ALS 25	48	22
ALS 21	48	14
ALS 19	49	14
ALS 22	38	68
ALS 17	48	13
ALS 24	39	22
ALS 18	51	16
ALS 36	50	100
ALS 34	58	13
ALS 32	57	22
ALS 33	57	94
ALS 35	48	68
ALS 53	69	95
ALS 51	34	35
ALS 52	66	92
ALS 54	59	86
ALS 50	23	14

Instructor Guided Group of Participants

Participant	time spent	VR Exam Score
ALS 41	39	62
ALS 39	44	56
ALS 37	46	100
ALS 42	46	67
ALS 75	46	94
ALS 38	42	100
ALS 43	40	100
ALS 48	50	100
ALS 46	40	68
ALS 44	42	99
ALS 45	41	14
ALS 47	44	13
ALS 54	40	86
ALS 55	36	22
ALS 56	41	100
ALS 57	38	52
ALS 59	38	56
ALS 60	44	13
ALS 61	60	13
ALS 62	40	56
ALS 63	43	94
ALS 64	45	100
ALS 65	41	100
ALS 66	40	100
ALS 67	43	100
ALS 68	46	100
ALS 69	42	95
ALS 71	34	22
ALS 72	36	14
ALS 74	83	100
ALS 76	38	11
